# Medico-Chirurgical Transactions, Published by the Royal Medical and Chirurgical Society of London, for 1836

**Published:** 1837-07

**Authors:** 


### Art. IX.

*Medico-Chirurgical Transactions, published by the Royal Medical and Chirurgical Society of London, for* 1836. Vol. XX.—*London*, 1837. 8vo. pp. 402.

The present volume commences by a communication from Dr. Yelloly, "*On Vascular Appearances of Mucous and Serous Membranes, as indicative of Inflammation.*" Its author, in a previous paper, performed a most important service to pathology, by showing, with respect to the mucous membrane of the stomach in particular, the little dependence which can be placed on vascularity or extravasation as an indication of the presence of inflammation. In this country, few would now be disposed to admit that inflammation had existed, unless some of its known products were brought forward as proofs. It is, however, true that the vagueness of the term, and particularly its application to a mere fulness of the small vessels, has been the chief cause of some of the theories which may be said to have disfigured, if not disgraced, medical science during the present century. Considerations of this kind, more than the novelties of Dr. Yelloly's paper, recommend it to notice; for the attention can scarcely be too often recalled to the consideration of facts which are of very extensive application.

It appears probable that a vascular condition of the spinal column has been "sometimes regarded as imparting a certain character of spinal inflammation to some diseases of an obscure nature, as tetanus, which did not actually belong to them." Dr. Yelloly has examined the spine of malefactors recently after execution; and he concludes, from his observations, "that a florid vascularity, communicating a scarlet hue to the whole column, is nothing more than venous turgescence, accompanied by slight extravasation." Dr. Yelloly has well alluded to the changes which occur in inflamed parts after death.

"Where inflammation has existed *externally*, death makes a very important alteration of appearance. The redness goes off, except where effusion has taken place, and the remains of it are seen; while the tumour sinks, except in as far as any deposition in the cellular membrane has taken place, and may render it permanent. But, in the *internal* organs, and more particularly the abdominal viscera, various modifications take place, from the peculiar situation which these parts occupy in the animal body. Being nearly connected with a double series of venous structure, that of the liver and that of the heart, in which a considerable portion of the blood is concentered after death, a retarding or obstructing cause operates on the veins which belong to them, and which, added to the softness of texture, very generally produces more or less fulness of the vessels; and thus an appearance of more or less vascularity, both on the external and internal surfaces of the chylopoïetic viscera." (P. 9.)

We recommend the conclusion of Dr. Yelloly's paper to our younger readers: it contains very judicious strictures on some of the continental notions of inflammation.

Mr. J. G. Perry has related a "*Remarkable Case of Varicose Aneurism, with Observations.*" The peculiarity in this case consists in the circumstance that the communication between the artery and vein was established independently of any external wound. We subjoin a short analysis of it. J. Allum, æt. 47, of delicate health, first recognized a small swelling below the left knee, which gradually increased. Two years after the commencement of this swelling, his wife first discovered a *palpitation* in the middle of the left thigh, which increased. Allum came under Mr. Perry's care in February, 1834. The swelling beneath the knee was a distinct aneurism. In the upper two-thirds of the left thigh there was a visible pulsation along the course of the femoral artery and vein, ending where the vessels pass through the triceps tendon. In and around the swelling was a distinct *frémissement*, which continued during the intervals of the arterial pulsations. On one examination, the frémissement was accidentally stopped by pressure with the finger upon the artery, just where it passes through the triceps; the circulation continuing in the vessel. A pad fitting to a spring was applied to this part. In September, 1835, the tumour beneath the knee remained as before; but the frémissement in the thigh was less in force and extent, and could not, as previously, be *seen*. The popliteal swelling now suddenly increased, the skin appeared ready to slough. The femoral artery was exposed, and found to be in a very diseased state. A ligature was passed round it; but the patient died of hemorrhage from the wound on the sixth day after the operation. We should mention that it was deemed unjustifiable to tie the femoral artery in an earlier stage of the case, and that this means was reserved until life was threatened by the increase of the popliteal tumour.

The state of the parts observed after death was as follows: The external iliac arteries, and especially the left, were very tortuous, being reflected upon themselves. The coats of the femoral artery, throughout its whole course, were scarcely thicker than those of a vein. Immediately below the origin of the profunda, the vessel was much dilated: its coats were softened and thin, and anteriorly was an aperture, from which the hemorrhage had taken place. The ligature had been placed at a very short distance below this part of the vessel. At the spot in the thigh where the communication had been presumed to exist between the artery and vein, there was an aneurismal sac, about as large as half a walnut, firmly ossified within; which, by the pressure which it had exerted upon the vein, had caused absorption of its coats, so as to form a circular opening, about two lines in diameter, into which the aneurism had burst; thus inducing a free and persistent communication between the vessels. Just below the aperture, the vein was obliterated at a single *point*, below which it was again pervious. In all the rest of its course up the thigh, it was diminished in size and thickened. There can be no doubt that the obliteration of the vein was an effect of the pressure of the pad. At the lower part of the popliteal artery, the vessel was dilated into a large aneurismal sac.

Mr. Perry has been able to find no other case of such an accident occurring in the arteries of the extremities; and, in addition to the knowledge which we at present possess of the little liability of veins to partake in ulcerative or sloughing process going on in contiguous parts, he quotes two remarkable illustrations. As explanatory of the vast complication of disease in the foregoing case, the anatomical condition of the parts is alluded to: "the vessels being enclosed in a sheath so dense and unyielding as to preclude the possibility of the vein undergoing displacement."

Mr. Sampson's "*Case of Recovery from the Insensibility of Intoxication, by the performance of Tracheotomy,*" is of great interest, and very creditable to him as a scientific surgeon. In a state of profound coma, when, after the ineffectual employment of the ordinary means, there appeared to be no hope of life, Mr. Sampson determined on the performance of tracheotomy. The operation was attended with complete success. It is peculiar in this case that there were "violent efforts of the extra-respiratory muscles," with impediment to the entrance of air from "collapse of the glottis, which (says Mr. S.) might be accounted for by a paralyzed state of the eighth pair of nerves and recurrent branches."

The perusal of this case leads us to ask Mr. Sampson whether there might not have been some foreign body, vomited during a previous stage of the intoxication, lodged at or beneath the glottis? It appears to us possible that such may here have been the case, both because the symptoms, as far as they are mentioned, accord with such a supposition, and because it is, we believe, contrary to what has been observed in such cases for so considerable a paralysis of the recurrent nerves of the par vagum to be associated with so much capability for the performance of their other functions, as would convey the stimulus efficient for a "violent effort of the extra-respiratory muscles."

The next paper, by Mr. Stafford, "*On the Treatment of Injuries received in Dissection,*" does not add much to the amount of our knowledge of such injuries. The accumulation, however, of authentic cases, particularly when the treatment has been followed by recovery, must be one of the means by which eventually we shall acquire correct views of the whole history of the accident in question; and Mr. Stafford has made a useful addition to our previous stock. There appears to be sufficient ground to conclude that in one, if not in two, of the cases here related, the infection took place without previous abrasion of the cuticle. But we must confine our notice simply to the practical part of Mr. Stafford's remarks.—The nitrate of silver should be rubbed immediately above the limit of the inflammation, sufficiently to blacken the cuticle. Violent pain in the wound, and symptoms of great constitutional disturbance of an irritative character, are best combated by muriate of morphia, repeated in sufficient quantities to keep up its effect. It is scarcely necessary to add, that general bleeding can most rarely be otherwise than injurious. But, if the inflammation in the hand and absorbents be very great, the patient has but little chance of recovery without the use of topical bleeding. Leeches should therefore be applied on the inflamed part, on any swelling arising from the wounds along the course of the inflamed absorbents, and on the glands of the axilla. The good effects of this treatment are sufficiently illustrated. When a swelling is formed, and it is doubtful whether or no it contain pus; when, together with it, there are pain, depression, fluttering pulse, delirium, exhaustion, and anxiety, the swelling should be opened; and, as the chief object, if matter be not formed, is to relieve the tension of the part, the incision should be of considerable length. The experience of many surgeons has established the propriety of the early opening of such swellings. As an example of the evils attendant on a neglect of this practice, Mr. Stafford remarks that, "in most of the cases related by Dr. Duncan, Colles, and others, where a swelling or abscess took place without an opening having been made into it, the patients did not recover."

The following inference appears to be fully justified by recorded facts, "that free incisions made into swellings arising from the absorption of animal poison are attended by the most beneficial results, and that they ought to be made in the earliest stage of their formation."

The following "*Case of Fracture and Displacement of the Atlas*, by Benjamin Phillips, Esq.," although "practically unimportant from its extreme rarity, is not wanting in importance as an example of the extent of injury which may be experienced by this portion of the spinal column, without harm to the important organ whose natural protector it is, or even to the economy; and, as a reason for rescinding the dicta that 'a fracture of the processus dentatus proves instantly fatal,' and 'that a fracture of the cervical vertebræ, (above the third,) with considerable displacement, is almost immediately fatal.'" W. Cross fell from a hay-rick upon his occiput, and was stunned. He recovered, walked half a mile to be bled and purged by the parish surgeon. For a month after the accident, when Mr. Phillips first saw him, he complained of "stiff neck," or inability to rotate the head. At the back of the neck, over the second cervical vertebra, was a small tumour. All the functions of the economy, excepting the rotation of his head, were well performed. He was treated on the supposition that an inflammatory action existed in the swelling of the neck, consequent upon the injury; but the only benefit derived was a diminution of tenderness.

About six months after the accident, a change in the character of the voice and a difficulty in swallowing led to a careful examination of the throat, and the detection of a slight fulness at the back of the pharynx; a circumstance which appeared quite reconcileable with the previous diagnosis. He soon afterwards had an attack of pleurisy, and this was followed by anasarca and hydrothorax, with which he died. Up to the last week he walked to the water-closet, and was never assisted in taking his food; and there was no evidence of motion or sensation being impaired, otherwise than has been mentioned. The neck only was examined after death.

"The condyles of the occiput still rested upon the articulating surfaces of the atlas, but the atlas was found to be, not a separate and independent vertebra, but an appendage to the axis. So much of its anterior portion as includes the surfaces by which it is articulated with the occiput and with the axis, had been violently separated from the posterior portion of its ring, and had been carried in an oblique direction, downwards and forwards, until it arrived upon the same plane, but anterior to the axis, to the body and transverse processes of which it became attached by perfect bony union; whilst the posterior fragment had suffered no displacement. Under these circumstances the bone presented two spinal foramina and four transverse, but no odontoid process. This organ having been fractured and separated, no organ passed through the anterior spinal foramen." (P. 82.)

By the oblique direction of the force which caused the fracture acting upon the atlas, the anterior portion was propelled downwards and forwards, between the axis and the pharynx, the posterior portion remaining as nearly as may be *in situ*. Thus, either the odontoid process must have been fractured or its transverse ligament torn; the occurrence of the former saved the patient's life. When the examination was made, there was scarcely any appearance of condensation of the surrounding tissues, or indeed any thing to warrant the idea that the region had been the seat of so much violence. There was no lesion of the spinal cord.

The advantages to be derived from *artificial Respiration in some Cases of Poisoning with Opium* are illustrated in the next communication, from C. J. Smith, Esq.—Jane H., æt. 25, at six in the morning, swallowed opium. The stomach-pump was employed, together with the ordinary means in such cases, but without success; so that, at 11½ A.M., as there was no pulse at the wrist, and only slight irregular action of the heart, and the respiration had nearly ceased, it was determined to employ artificial respiration, by means of a pair of bellows through the nostril. This was continued for an hour, without intermission, at which time the heart seemed to be rallying; but, if left to itself, it sank again. Other stimulants were employed, and the treatment continued till two P.M., when she was so evidently rallying that it was deemed safe to leave her. At three P.M. she was found relapsing into her former state. Artificial respiration was again employed, and continued for two hours; when it was suspended on account of the pulse having become regular, and her having made some attempts to move, and showing evidence of pain on being pinched. She perfectly recovered.

This case (and it is not a solitary one of the benefit attending such treatment, the sixth volume of the "London Medical Observations and Enquiries" containing a similar instance, recorded by Mr. Whately,) shows that, by keeping up artificial respiration, the vital powers may be maintained, until the deleterious influence of the poison is worn out or exhausted; probably, as has been suggested, by its becoming decomposed: and it affords the strongest encouragement for the assiduous employment of a remedy, which may effect a cure when all other means have been tried without effect, but which does not appear to have been used so frequently as its merits demand.

Dr. P. N. Kingston's "*Remarks on two Forms of Atrophy of the Heart's Valves, which interfere with their Functions,*" need not detain us long. Both forms of this deficiency have been previously noticed; and, as Dr. Kingston does not bring forward any satisfactory method of diagnosis or of treatment, we shall merely allude to his anatomical description of the disease in question, which may be useful to some of our readers. "The lesion consists in a simple shortening of the mitral or tricuspid valves, without any diminution of its natural thinness, pliancy, and transparency; the orifice to which it belongs possessing at least the ordinary caliber. The extent of wasting is often considerable. Thus the length of the posterior lamina of the mitral valve was in one case reduced to three lines; in another, it nowhere exceeded a line and a half. The laminæ of the tricuspid valve are naturally from eight to eleven lines in length; but in one case they all three fell very far short of this, and one was only three lines." Another lesion closely allied to this is "the interruption of the continuity of a valve by apertures, sometimes of large size, and sometimes so numerous as to reduce the structure to a mere network, while the remainder is often very attenuated, especially towards the edges of the apertures. Sometimes a large gap is seen, subdivided only by a few thready fibres. These, in the case of the auriculo-ventricular valves, are generally prolongations of chordæ tendineæ." (P. 91.) In about thirty of the cases of diseased valves, of which Dr. Kingston took notes during a definite period, one or other of these species of atrophy existed in ten. From what is now well known of the effects of impediments to the heart's physiological condition existing in any of its orifices, it is unnecessary for us to follow Dr. Kingston in his reasonings on this subject.

False inferences may have been drawn as a consequence of this defective state of the valves having been hitherto but little regarded, and, on this account, the following remarks are worthy of attention.

"When there have been post-mortem examinations of cases in which this lesion existed, but in which it passed unobserved, the symptoms to which it had given rise during life have necessarily been referred to some other cause. Thus, if this lesion had communicated to the ear a morbid murmur, the case has been considered as affording an instance of morbid murmur independent of valvular disease. Whenever it has occasioned or helped to occasion palpitations, syncope, dyspnœa, dilatation of the heart's cavities, or dropsy, some concomitant or hypothetical lesion must have unjustly bore the blame. Hence have been deduced erroneous conclusions respecting the nature of diseases, and the diagnostic import and value of symptoms, which a knowledge of these lesions will rectify." (P. 109.)

Mr. Benjamin Travers, Jun. has added another case to those of *dislocated Thigh-bone* "termed unusual, because not hitherto described in any systematic work upon the subject." The individual fell from a height of about twenty feet, the left buttock striking upon a coil of chain-cable. The limb was dislocated, but was not reduced. Eight months after the accident, "the left buttock is flattened; the trochanter rather below, and to the outer side of the anterior and superior spinous process of the ilium. The neck of the bone lies apparently between the two anterior spinous processes. Its head cannot be felt; it is invested by an abundance of bony matter, which extends backwards and inwards over the brim of the pelvis and iliac vessels, occupying in front nearly the whole space between the inferior spine of the ilium and that of the pubis respectively. There is complete eversion, slight mobility, and imperfect progression with the aid of a crutch." In addition to the species of dislocation hitherto described in systematic works, Mr. Travers considers from this and other cases which are recorded, that it may now be further admitted that the head of the bone may assume a position either directly *above* or *below* the articular cavity.

A communication by Sir B. C. Brodie, on "*Injuries of the Spinal Cord,*" will well repay a careful perusal. Wounds and injuries of the head have largely attracted the attention of surgical writers: the same cannot be said of similar lesions of the spinal cord, and the intent of the present paper is to remedy this defect in surgical literature. Our limits will not allow us to lay before our readers as much of this paper as we could wish. On the subject of wounds, Sir B. Brodie has nothing new to communicate. The conditions found on dissection after injuries of the spine are thus classed: 1. Fractures of the vertebræ, without displacement of the fractured surfaces. 2. Fractures with depression or displacement of bone, diminishing the diameter of the spinal canal, and occasioning pressure on the cord. 3. Fractures complicated with dislocation. 4. Dislocations not complicated with fracture; the possibility of which is now fully established. 5. Extravasations of blood on the surface or membranes of the cord, rarely of any considerable extent. 6. Small clot of extravasated blood in the substance of the cord. 7. Laceration of the cord and its membranes, very various in kind and degree. 8. Lesion of the minute organization of the cord from a blow, without fracture or dislocation, and where the investing membranes are apparently in no way affected. In such cases, if the spinal cord be examined at a very early period after the accident has occurred, the central part is softer than usual. If the patient survive for a longer period, this disorganization extends. It is the most common consequence of injuries of the spine, and the cause of the worst symptoms in the majority of cases. In concussion of the brain, Sir B. Brodie has already remarked that we are not justified in the conclusion that, because no changes are to be detected after death, "there is therefore, in reality, no organic injury;" and that "it is difficult to conceive in what other manner concussion of the brain can operate, so as to produce the effects it is known to produce: and, if we consider that the ultimate structure of the brain is on so minute a scale that our senses are incapable of detecting it, it is evident that there may be changes and alterations in it which our senses are incapable of detecting also." These remarks he here applies equally to cases of concussion of the spine.

The nature of the injury of the spinal cord, which, in cases of violent concussion, at once impairs or even destroys its functions, is a curious subject of enquiry. The author suggests, but has never practised, the experiment of macerating in alcohol a spinal cord so contused, and then examining its fibres to detect in what respect they are changed from their natural condition. For the following reasons, it appears doubtful whether the softening and dissolution of the cord after injuries is inflammatory; because, 1, this softening can sometimes be detected before there could have been time for inflammation to have produced it; 2, the softened part at first exhibits no vascular appearance; 3, when the cord is softened to disorganization, the investing membranes are mostly natural in appearance, shewing none of the products of inflammation; 4, the symptoms which mark the progress of these changes are merely a continuation of those which the concussion of the spinal cord has occasioned in the first instance, and which must have been wholly unconnected with inflammation. This softening is regarded by Sir B. Brodie as resembling that described by Rostan as occurring in the brain. Of course, inflammation may follow these injuries, and the possibility of its occurrence must not be overlooked.

The symptoms attending injuries of the spine will vary according to the situation, the kind and degree of injury, and according as (from circumstances unknown to us,) life is more or less prolonged. Sir B. Brodie has analyzed the associated symptoms, and has endeavoured to point out under what circumstances each symptom presents itself. We must confine ourselves to such parts of his observations as are more or less generally known; not without regret, for there is in his observations so little that is superfluous, that we could, had we space, willingly record them all.

*Paralysis of Voluntary Muscles.* The lower limbs are more frequently paralyzed than the upper, even where the lower part of the cervical spine has been injured; some of the origins of the brachial plexus being probably still above the injured part. This circumstance is remarkable, as it is contrary to what happens when the functions of the spinal cord are interrupted in consequence of caries of the cervical vertebræ. In these last cases, the paralysis is often complete in the upper limbs for many weeks, or even months, before it extends to the lower. Paralysis of the upper limbs has been known to follow contusion of the dorsal vertebræ; but it is probable that such cases were but apparent exceptions to the general rule of paralysis being confined to the parts below the injured portion of the spinal cord. It is easy to suppose that, in such cases, a contusion has taken place above the obvious seat of injury. We think that Sir B. Brodie is not justified in concluding that *muscular spasms* are in all cases the effect of pressure, when accompanying injuries of the spine. This conclusion is founded on a very limited number of cases, and the whole history of spasmodic affections, as well as of the inferences to be derived from what we know of the effect of poisons, must make us hesitate in agreeing with the author on this point.

*Affections of Nerves of Sensation.* It is well known that there are in these injuries the same varieties with respect to sensation that there are with respect to muscular action. When recovery takes place, the restoration of sensibility usually precedes that of voluntary motion.

*Affection of the Respiration.* Artificial respiration would be the means of prolonging life for several hours, where the spinal cord is divided above the phrenic nerves. As in the case communicated by Mr. Phillips, dislocations of the first and second vertebræ do not prove fatal in every instance, in the manner generally supposed. Sir B. Brodie relates the case also of a child, where the transverse ligament of the axis had given way, and the odontoid process projected considerably into the spinal canal. The dura mater was entire, and prevented the dislocation being so complete as it would have been otherwise. The child died with symptoms of hydrocephalus. The respiration, when performed only by the diaphragm, seems to be insufficient to maintain life, and recoveries under such circumstances are very rare. The lower the injury is in the back, the less is the respiration affected; and, wherever the injury is situated, a disposition to cough, with a copious expectoration, is likely to occur some time after the accident.

*Priapism.* The author has never known this to occur excepting there was paralysis. "It seems," he says, "to be connected with injuries of the upper rather than with those of the lower portions of the cord: at least, I am not aware that I have met with it where the seat of the injury has been below the sixth dorsal vertebra. It occurs even when the sensibility of the parts is totally destroyed, and may be induced by the mechanical irritation caused by the introduction of a catheter, where the patient is entirely unconscious of the operation." (P. 140.)

*Affections of the Urinary Organs.* To the changes produced in the secretion of the kidneys and bladder, in injuries of the spine, Sir B. Brodie has, since 1807, paid particular attention; and his description of them we shall here give in an abridged form.

"The first effect of a severe injury of the spinal cord is not unfrequently to occasion a marked diminution in the quantity of urine secreted. This is most observable when the injury is in the lower part of the neck, and where, in consequence, the function of respiration is very much impaired. . . . The same thing, however, may occur when the injury is in the lower part of the spine. . . . In some cases the urine which is first secreted after the occurrence of the accident . . . has a peculiarly offensive and disgusting odour. In others it is highly acid, having an opaque yellow appearance, and it deposits a yellow, amorphous sediment." . . . But most commonly "the urine is voided of an ammoniacal odour and turbid: when allowed to cool, it deposits a large quantity of adhesive mucus, and is highly alkaline. After some time a quantity of white matter (phosphate of lime,) may be detected in the mucus, and it is tinged with blood. At a still later period, a considerable quantity of coagulum of blood is blended with the mucus and urine. These appearances very commonly shew themselves as early as the second or third day after the occurrence of the accident; sometimes between the seventh and ninth days. I have not observed that injury of one part of the spine is more liable to produce them than that of another. There is a great variety in the period of their duration. In fatal cases, they sometimes continue to the last; at other times, they continue for two or three weeks, then subside, and the urine remains transparent and of an acid quality afterwards. In other cases, the quality of the urine varies almost from day to day, without any manifest reason for the change." (P. 142 *et seq.*)

The adhesive mucus above mentioned is furnished by the bladder and ureters; a consequence of inflammation occasioned by the spinal injury. The effects of this inflammation are very manifest on dissection.

*Alteration of the Vital Temperature.* In one case in which there was a separation of the fifth and sixth cervical vertebræ, and where respiration was performed by the diaphragm only, the temperature between the scrotum and thigh was 111° F. This is mentioned as confirmatory of the experiments of Chossat, which tended to prove that there was a remarkable evolution of animal heat in animals, on division of the superior portion of the spinal cord. Sir B. Brodie has seen several cases in which an accidental injury of the spinal cord has produced the same effect.

The disposition to *gangrene* in these cases is an evident consequence of the injury of the cord, as it occurs whether the heart's action be strong or feeble, and is limited to the parts below the injury. The sloughs sometimes commence the second day after the accident, when it has affected the cervical portion of the cord.

The reaction which follows the collapse that immediately attends upon severe injuries of the cord is such as indicates general debility, rather than active inflammatory disease. "Inflammation of the membranes of the spine," says the author, "is undoubtedly a much more rare consequence of injuries of the spine, than inflammation of the membranes of the brain is of injuries of the head."

The symptoms of injuries of the spine, and the results to which they lead, appear to be characterized by no material difference, whether the cord be lacerated or compressed, or has undergone that kind of disorganization which is produced by a severe concussion; and the great majority of the symptoms are the same, whatever part of the spinal cord has suffered from the injury. There is only one order of symptoms with respect to which a great difference exists, according as the seat of the injury is in one or another part of the spinal cord: i. e. those connected with respiration. A consideration of the connexion between the various parts of the cord and the muscles of respiration will explain why the danger to the patient's life is greater and more imminent in proportion as the injury is nearer to the brain.

*Treatment of Injuries of the Spine.* Any attempt to replace a dislocated or fractured bone of the cervical vertebræ must be made with the greatest caution, if undertaken at all: in the lower part of the spine, the attempt at reduction may not only be made with impunity, but with success. Both theoretically and from the results of the practice, as far as it is known, Sir B. Brodie is adverse to trephining the spinal column. The supine and horizontal posture on a mattress is proper. If inflammation of the membranes occur, it should be treated by bleeding, which may require to be repeated. But the indiscriminate employment of bleeding in such cases is strongly condemned, as might be supposed from the views as to the pathology of the disease which we have already noticed. A combination of ammonia with purgatives often facilitates their operation.

Before noticing the "*Observations on some Tumours of the Mouth and Jaws*, by R. Liston, Esq.," we must object to the extremely loose and careless manner in which they are communicated. A sentence, at page 170, commencing "Lodged deeply amongst the bones of the face," &c. is a rare example of bad punctuation, incorrect grammar, and confused construction; and the paper is disfigured in other parts in a similar manner, though less in degree. "Disease repullulating from parts which surrounded the original nidus of the mischief," (p. 179,) appears to us a very good specimen of catachresis; a species of metaphor which requires but to be expressed in sober Saxon to manifest its absurdity. We allude to these points, because they diminish the value of Mr. Liston's communication; and because, as he is likely to continue an active contributor to the stores of surgical science, we should wish to be able to derive pleasure as well from the manner as the matter of his future communications.

One principal object of the present paper is to point out the characters of those tumours of the jaws which may with safety be removed. We must content ourselves with a description of a tumour of the jaw, which, in several instances that have fallen under Mr. Liston's notice, he has removed with success. He objects to attempting the removal by operation of those which are malignant, unless the case is met with in its commencement. He has recorded the case of one individual operated on thus early, in whom the tumour was of a decidedly brain-like character, presenting a smooth greasy section, broken up and bloody-looking at a part where it was attached to a decayed molar tooth. A period of four years has elapsed since the operation without a return of the disease.

The following is Mr. Liston's account of the tumour admitting operation.

"The simple tumour, which, whether involving the upper or the lower jaw, differs in consistence and in form also from those soft, lardaceous and brain-like masses whose appearance and progress I have shortly alluded to. They attain, though slowly, a great size; they present a globular or botryoidal form, displace the surrounding soft and hard parts, project from the countenance, and, deranging the features, produce great and frightful deformity. The skin may be thinned and pervaded by enlarged venous branches; it is discoloured, but not incorporated even at an advanced stage with the morbid mass, nor are any of the surrounding tissues contaminated. The projection towards the mouth . . . . is hard and elastic, and conveys the feeling of brawn, interspersed with bony particles; but it is covered by a continuation of the mucous lining of the cavity slightly thickened and altered, furnishing an inconsiderable discharge, and that neither offensive nor of a bad quality. This growth in the mouth presents indentations made by the teeth, with which it comes in contact. The hard palate, when the upper jaw is involved, is generally covered by a thick layer of the tumour, which projects over and lies in contact with, but is not adherent to it, nor to the gums supporting the teeth of the opposite side." (P. 171.)

Mr. Liston has found few such tumours described as affecting the upper jaw. The paper contains an account of the method of operating for the removal of these morbid growths. We have not space to do it justice, but those who are aware of the great operative skill of Mr. Liston will refer with confidence to his opinion on this subject.

The paper by Dr. John Burne, "*on Inflammation, Chronic Disease, and perforative Ulceration of the Cæcum*," is valuable, and will be very acceptable to many of our readers. The attention of the profession has of late years been considerably directed to the disease in question; and we are surprised to find Dr. Burne stating that he has not met with any notice of it except in detached cases in the Medical Journals. In our Second Number (p. 501,) we gave an account of three separate publications on this subject, and referred to several others. However, as the disease is still too little known, and as Dr. Burne's account of it is very good, we shall shortly extract from his paper, its main characters, diagnosis, and treatment.

This affection of the cæcum is of frequent occurrence, about twenty cases having fallen under Dr. Burne's observation. They are, says Dr. Burne, apt to be confounded with idiopathic abdominal inflammations, and to be treated as such, much to the injury, if not to the destruction, of the patients. In all Dr. Burne's cases, the disease has been symptomatic of some mechanical exciting cause, as the lodgment of undigested food, of fruit stones, or of concretions, which the structure of the cæcum and appendix favours; and hence the peculiar features of the disease. Before describing the disease, we must say that we see no reason, even from Dr. Burne's own cases, to conclude that it is *always* occasioned by a mechanical cause.

"The peculiarities of this inflammation," says Dr. Burne, "produced by such mechanical causes, are, the marked and fixed local signs; the invasion of them without any obvious cause while the patient was in health; their gradual development; their obstinacy; the late supervention of the febrile movement, and its less degree in proportion to the local affection and suffering, and the less anxiety depicted in the countenance than is noticed in the idiopathic enteritic inflammation. These are the peculiarities." (P. 202.)

The development of the symptoms has been, in all Dr. Burne's cases, in the following order: uneasiness followed by pain deeply seated in the right ilio-inguinal region, arising unexpectedly whilst the person was in health, and not preceded by rigor or exposure; the pain increases, is fixed and constant; tenderness, fulness, and tension of the whole ilio-inguinal region follow; the bowels are constipated, and do not reply to medicine, and the patient grows sick and vomits. Some fever now manifests itself; the pulse has a character of irritation and inflammation combined; the patient lies on his back quite still. This condition may persist for several days; the fulness and tension extending to other parts of the abdomen, and the abdominal parietes covering the cæcum become *exquisitely tender*. The constipation continues, but the vomiting does not become frequent and distressing as in enteritis. A subsidence of the symptoms rarely takes place in less than seven or eight days. Sometimes the patient sinks without relief of symptoms. At others, an emphysematous tumour occurs, which proves to be a fæcal abscess.

"The *diagnosis* of these cases is determined with precision by the seat of pain, the exquisite tenderness and the tension; by the sudden invasion of the symptoms whilst the patient was in health; by the local signs preceding the febrile movement; by the degree of fever being less than in idiopathic inflammation; and by the less marked anxiety of countenance." . . . "The circumscribed fulness and hardness in the region of the cæcum will give assurance of the seat and nature of the affection." (P. 205.)

In the *Treatment* of this disease, it must be remembered that the cause of the inflammation is mechanical in many cases, and that the first object is to remove this cause. The indications are to moderate the inflammatory action to a degree consistent with the vitality of the organ affected, and to accomplish this with as little expense to the bodily powers as possible; so that, if fæcal abscess should form, nature may be able to go through the tedious and difficult process which would be inevitable. Bloodletting must be moderately practised. Warm thin poultices must be applied after leeches. Common enemata and aperients must be employed to dislodge the offending matter. Fomentations are very useful. If the symptoms persist, and an emphysematous tumour presents in the lumbar or ilio-inguinal region, it should be immediately opened by a free incision. Great attention will now be required to support the patient's strength.

In addition to the above-described acute inflammation, the cæcum is not unfrequently the seat of a subacute chronic inflammation or pathological congestion, which induces thickening of its tissues and contraction of its natural capacity, eventually determining, as in the case of stricture elsewhere, a complete and fatal obstruction. The effects produced by perforative ulceration of the appendix cæci depend much upon its situation. "If," says Dr. Burne, "it should happen to depend into the pelvis, then the pelvic viscera will be implicated: if it is situated upon the iliac fascia and beneath the cæcum, then the belly of the iliacus internus and neighbouring adipose tissues will be involved, and the course of the abscess be determined accordingly." Small bodies are not uncommonly found in the appendix, and the only effect they produce may be ulceration of the mucous membrane of that organ; but, if the substance be larger than the canal of the appendix, perforation is the ultimate consequence. When the peritoneum becomes perforated, the inflammation increases, and either a general peritonitis is produced, or the inflammation may be limited to the vicinity of the perforation, and an abscess may form. This abscess may come forward and burst, and the patient recover; or it may remain circumscribed and stationary, forming a deep-seated painful tumour in the neighbourhood of the cæcum, which, by its proximity to this gut, may produce obstinate constipation and sympathetic disturbance of the whole system, and thus gradually wear down and exhaust the patient. Of these various forms of disease, Dr. Burne has recorded some cases in detail.

The attention of Dr. Thomson, of Edinburgh, and that of his son Dr. William Thomson, has for some time been given to the subject of the next communication by the latter gentleman, "*On black Expectoration and black Matter in the Lungs.*" Dr. W. Thomson is desirous "of calling the attention of the profession to the evidence in favour of the extraneous origin of the black matter by which the sputa are liable to be discoloured and the lungs to be infiltrated, which has been supposed to be derived from the occurrence of these affections in persons who, from their occupations, are particularly exposed to the inhalation of carbonaceous powders or gases, such as coal-miners and moulders in iron-works." The present paper is the commencement of the enquiry, and consists of cases and communications from some individuals who have had considerable opportunities of observing the disease. Our notice of it must necessarily be very short. The cases are thus arranged: I. Those in which the lungs have actually been found infiltrated with black matter after death; *a*, with symptoms of pulmonary affection during life, the expectoration being for a longer or shorter time of a black colour; *b*, with no pulmonary ailment, or at least with no black expectoration during life. II. Those in which the disease was suspected to exist from the nature of the symptoms, but which were not examined after death. Of I. *a*, ten cases are known, of which nine occurred in persons engaged in coal-mines, and one in a moulder employed in the Carron iron-works. Of the second subdivision, six cases have come to Dr. W. Thomson's knowledge, all in colliers. Numerous cases of the second class are recorded, as well as some very interesting communications from practitioners residing in coal districts. We subjoin a description of the principal anatomical character of the disease from one of the recorded cases.

"When cut into, both lungs presented one uniform, black, carbonaceous colour, pervading every part of their substance. The right lung was much disorganized, and exhibited in its upper and middle lobes several large irregular cavities, communicating with one another, and traversed by numerous bands of pulmonary substance and vessels. These cavities contained a good deal of fluid, which, as well as the walls of the cavities, partook of the same black colour. A considerable portion of the pulmonary substance surrounding them was dense, hepatized, and friable. The rest of the lung was somewhat condensed, and very œdematous. The serum, when expressed, was of the same black colour as the substance of the lung. Some minute hard points could be felt in various parts of both lungs, but they did not differ at all in colour from the surrounding substance, and no distinct tubercular deposition or infiltration could be detected in those portions of the lungs which were most hepatized, even with the aid of the microscope. The texture in these parts appeared quite uniform, and the minute hard points felt in other parts rather conveyed the impression of their being merely the ends of small bronchial branches divided on making the section. The bronchial glands did not appear enlarged, but partook of the same black colour as the substance of the lungs." (P. 240.)

With the exception of the occurrence of the black sputa, which has been stated not to exist in all cases of the disease, it does not appear to possess any very characteristic symptom. Early dyspnœa existed in many cases; but some are mentioned as resembling asthma and chronic mucous catarrh and chronic bronchitis. A great variety of opinion has existed, and still continues to exist, as to the origin of the disease; the framers of such opinions having been influenced apparently by the circumstances in which the cases that fell under their notice appeared to originate. Thus, the disease has been ascribed to the inhalation of coal-dust, gunpowder smoke, lamp smoke, choke-damp, the impure air of mines, consisting of a mixture of carbonic acid gas and the atmospheric air; and one of Dr. W. Thomson's correspondents is disposed to consider that the black matter is generated in the lungs; adding, that "when its formation has once taken place, it appears never afterwards entirely to leave the lungs, but maintains its existence within the body during the remainder of life, and this although the individual afflicted with it does not continue to work as a miner." He speaks also of a woman who "has not been in the pit for fourteen years, and has had constant black spit, without any pectoral complaint." T. Ross, "after having been a number of years employed as a miner, was at sea for three years; and during the whole of that time his sputa were never perfectly free from a black impregnation. I could cite numberless cases similar to these." For assisting those who may be disposed to the prosecution of this enquiry, the following questions are subjoined.

"If such an appearance (i.e. considerable and long-continued black sputum,) has presented itself to your notice, we are desirous to be informed, 1. Whether it has been found in particular mines or manufactories more than others, and in one class of workmen in those establishments more than another? 2. Whether it appears to you to depend on an imperfect evolution of carbon from the lungs, in consequence of persons respiring a confined or vitiated atmosphere, or to be occasioned by the inhalation of extraneous carbonaceous matters; and, if by the latter, what you conceive these matters to be:—the smoke of the candles or lamps employed by the workmen; or of the gun-powder employed in some mines in blasting, or what other agent? Should you have formed any opinion on this subject, may we request you to state the grounds of it? 3. Whether the black expectoration occurs more frequently in persons apparently healthy, particularly as it regards the pulmonary organs, or in persons in whom there is reason to suspect a morbid condition of these organs, such as chronic bronchitis or tubercular consumption? 4. Whether the black colour of the sputa is constant or only occasional? 5. Whether, in any cases of this kind, you have had an opportunity of ascertaining, after death, the state of the lungs and bronchial glands? 6. Whether, in any dissections you have made of persons whose sputa had not been so discoloured, you have found the lungs infiltrated with black matter capable of tinging the hands and of communicating to water the colour of china-ink." (P. 299.)

This communication is highly creditable to the industry and talents of its author.

The next communication is the termination of a *Case of Ligature of the external Iliac Artery*, which was related by G. Norman, Esq. of Bath, in the tenth volume of the Society's Transactions. It contains an interesting account of the collateral circulation which was established subsequent to the ligature of the external iliac artery, the man having lived twenty years after the performance of the operation.

"*Researches on some points of the Pathology of Pulmonary Tubercles*, by P. N. Kingston, m.d." The design of the present paper is to show, 1, that the common pulmonary tubercle is a vascular texture; 2, that it sometimes originates in an alteration of the air-cells and their secretions; 3, that now and then it is entirely healed, even when it has extended over a very large portion of the lungs. In favour of the vascularity of tubercles, Dr. Kingston has offered the strongest evidence which he could be expected to do, and which we can scarcely contradict; for he says that he has seen red lines in tubercles, and that there was no room to doubt that they were blood-vessels.

"A careful section," says Dr. K., "being made of the tubercle intended for examination, the cut surface was seen by a strong magnifying glass, sometimes also by the naked eye, to be traversed by continuous red lines, which were sometimes short and unconnected, but often of considerable length, and making frequent ramifications and anastomoses, quite after the manner of small blood-vessels." . . . "These red lines could not be removed by being gently scraped with the edge of a scalpel. In many instances they might be traced from the centre to the circumference of the tubercles, whence they were sometimes seen to extend into the adjacent pulmonary parenchyma, or to communicate with the vessels in the neighbourhood." (P. 310.)

So great appears to be the facility with which Dr. Kingston can detect these red lines in tubercles, that one can hardly help feeling surprise, after reading his paper, that their vascularity should be a subject of dispute at the present day. And yet it is true that not only we ourselves, but the most eminent and experienced pathologists of past and present times, have failed in observing what seems to have been so readily discovered by Dr. Kingston. Is there not some fallacy in this?

On the second point, the origin of tubercle, it will be seen that Dr. K. maintains no novel opinion; and he has recorded a remarkable case, in which a tuberculous affection, so extensive as to have disorganized the whole of one lung and a fourth part of the other, had been healed."

The next paper consists of "*Observations on some of the Forms of Atrophy of Bone*, by Thomas Blizard Curling."

The term Atrophy is here applied to "all those changes (in bone) evinced by loss of substance, unaccompanied by any alteration in texture or organization, and without any reference whatever to the morbid action that produces it; since, in the majority of cases it is impossible to determine with accuracy whether the loss is the result of increased absorption or of defective nutrition. . . . Atrophy may occur in bones generally, or be limited to certain bones or to particular parts of bones." We need not allude to local atrophy as an effect of pressure. Atrophy may take place in a part or the whole of a bone, as a result of injury; so that, "without any evident alteration in external configuration, by a change affecting both hard and soft particles, the bone may be rendered smaller and of diminished weight." To this change our author applies the term *concentric atrophy*. The bones, as well as the soft structures, fade and waste away when their activity is diminished or their functions are suspended, *e.g.* bones of stumps after amputation, and those of anchylosed limbs. They waste likewise when deprived of nervous influence. It may be questioned, however, in the instances brought forward by Mr. Curling, how far the diminution of activity of the wasted limbs, and not the loss of nervous influence, was the cause of the atrophy. But the influence of the nervous system over capillary vessels is shown in a remarkable case recorded by Mr. Travers, "in which union between the ends of a fractured leg that was paralyzed from fracture of the lumbar vertebræ, failed to proceed; whilst the extremities of the humerus, fractured at the same time, united perfectly in the usual period."

Atrophy of soft parts is sometimes an effect of a diminution in the normal supply of blood; and Mr. Curling has adduced the following curious examples of a similar effect produced by a similar cause in bone. Nutrition is maintained in the long bones by the vessels passing from the periosteum and the proper nutrient vessels of the bone. It occurred to Mr. Curling that, in fractures of such bones, one part must have the supply which it derives from the nutritious artery entirely cut off; and, although both orders of vessels freely communicate with each other, yet the minute canals, through which the external or periosteal pass, being of a dense unyielding nature, these vessels must be prevented from undergoing that rapid increase in size which in the soft structures constitutes so efficient a provision for a due circulation. Mr. Curling accordingly examined sections of fractured cylindrical bones, in order to ascertain if the ends which had been deprived of their normal supply of blood from the nutritious artery underwent a corresponding degree of atrophy. The result of the examination bore out his previous reasoning. In femurs fractured below the entrance of the nutritious artery, he found the interior cavity of the inferior extremity enlarged, the cancelli expanded and the walls thinned; a form of atrophy to which he proposes to apply the epithet *eccentric.* A similar alteration was also observed in fractured tibiæ; whilst in the humerus, broken near the middle and somewhat above the entrance of the nutritious artery, the upper portion was the seat of this change.

This wasting is not *constantly* met with, and, if the above explanation be correct, it must not be expected, 1, in bones recently fractured; the process of atrophy being gradual: 2, In bones long united; because of the establishment of a collateral circulation; but, in old persons, and in those of weak powers, the circulation may never be completely reinstated, in which case the atrophy will be permanent: 3, In bones fractured during the period of growth. In proof of the fact, that atrophy occurs in the portion of a fractured bone from which its normal supply of blood is cut off, Mr. Curling refers to specimens in most of the museums in London; and he considers them as sufficient to prove that the *eccentric* atrophy was not accidental, but dependent on some uniform cause. The atrophy of old age, Mr. Curling states to be chiefly eccentric. Under the same kind of atrophy is here included the disease termed mollities ossium; which Mr. Curling concurs with others in regarding as the result of defective nutrition. He has presented a table of all the known cases which he has been able to find of this disease; which, together with some observations, may assist any one who is desirous of investigating the difficult subject of the pathology of this disease.

Mr. Birch next terminates the history of a case commenced in the thirteenth volume of the Society's Transactions; and the present concludes by the relation of an instance of *complete Recovery after the Removal of a Portion of Lung which projected from a Wound in the Thorax;* communicated by Wm. Forde, Esq.

Our readers will not fail to observe that this last publication of the Medico-Chirurgical Society is, on the whole, a valuable one; and we doubt not that, under its present management, it will continue to supply the profession annually with new and important information.

